# Healthcare for Trans*gender People in Germany: Gaps, Challenges, and Perspectives

**DOI:** 10.3389/fnins.2021.718335

**Published:** 2021-09-07

**Authors:** Nora Guethlein, Melina Grahlow, Carolin A. Lewis, Stephan Bork, Ute Habel, Birgit Derntl

**Affiliations:** ^1^Department of Psychiatry and Psychotherapy, University of Tübingen, Tübingen, Germany; ^2^Graduate Training Centre of Neuroscience, University of Tübingen, Tübingen, Germany; ^3^Emotion Neuroimaging Lab, Max Planck Institute for Human Cognitive and Brain Sciences, Leipzig, Germany; ^4^International Max Planck Research School on Neuroscience of Communication: Function, Structure, and Plasticity, Leipzig, Germany; ^5^Department of Psychiatry, Psychotherapy and Psychosomatics, RWTH Aachen University, Aachen, Germany; ^6^Institute of Neuroscience and Medicine, JARA-Institute Brain Structure Function Relationship (INM 10), Research Center Jülich, Jülich, Germany; ^7^LEAD Graduate School and Research Network, University of Tübingen, Tübingen, Germany; ^8^International Max Planck Research School for Cognitive and Systems Neuroscience, University of Tübingen, Tübingen, Germany; ^9^TübingenNeuroCampus, University of Tübingen, Tübingen, Germany

**Keywords:** transgender, transidentity, transsexualism, healthcare, internalized homonegativity, gender-affirmative healthcare

## Abstract

People whose gender does not correspond to the binary gender system, i.e., trans^∗^gender people, face two main problems when it comes to healthcare in Germany: (1) They often suffer from general psychiatric comorbidities as well as specific and significant mental distress due to gender dysphoria, and (2) the German healthcare system lacks sufficiently educated and clinically experienced medical personnel who are able to provide specialized healthcare. Aside from transition, it often is extremely difficult for trans^∗^gender people to get access to and be integrated into the medical system. Stigmatization and pathologization in treatment are widespread, as are long waiting times for specialized healthcare providers who are often only accessible to those trans^∗^gender people willing to travel long distances. Frequently, trans^∗^gender people face further difficulties and barriers *after* transition, as some healthcare professionals fail to provide suitable care (e.g., gynecological consultation for transmen). The ICD-11 German Modification (ICD-11-GM), which should be routinely used by 2022, implements a depathologization of trans^∗^gender people in the medical system. This paper compares the issues related to health and healthcare of trans^∗^gender people in Germany with those in other European countries. We review the care offered by specialized centers with regard to treatment of and support for trans^∗^gender people. We conclude with specific proposals that may contribute to establish an improved, up-to-date, gender-sensitive healthcare system.

## Introduction – Gaps and Challenges

Modern societies are widely dominated by a hegemonic binary view of people’s gender identity as well as a heteronormative understanding of relationships. Even in liberal democracies, where a pluralist understanding of different sexual, religious and lifestyle orientations are commonly accepted, trans^∗^gender people are confronted with this “heterosexual matrix” (Butler, 1991) on a daily basis. Correspondingly, the healthcare systems in these societies have institutionalized an exclusive binarity: medicine largely operates with the classification “male” and “female” as the only expected expression of gender, with most of the current models for mental disorders still relying on male data only (Shansky, 2019). This is especially problematic when it comes to healthcare for non-binary people. They face insufficient medical care, which is aggravated by treatment providers’ lack of awareness of their concerns and insufficient knowledge of gender-sensitive medicine. People whose gender identity does not correspond to the perceived norm are negatively affected by this lack of knowledge with some of them facing severe stress and discomfort. Unsurprisingly, trans^∗^gender individuals are at higher risk to report mental health problems than cisgender individuals. For example, a recent comparative study of mental health issues among cisgender and trans^∗^gender people indicated that 77% of the included trans^∗^gender participants were diagnosed with a mental disorder vs. 37,8% in cisgender participants (Hanna et al., 2019). Several studies show an elevated risk for affective disorders, anxiety disorders, and addictive disorders in trans^∗^gender people compared to cisgender individuals (Reisner et al., 2016; Bouman et al., 2017; De Freitas et al., 2020). In addition, increased suicidality for trans^∗^gender people compared to the cisgender population has been reported (Goldblum et al., 2012; Bailey et al., 2014; Reisner et al., 2016; Adams et al., 2017; Yüksel et al., 2017). This increased risk of comorbidities could be replicated in several countries worldwide, including data from the Lebanon (Ibrahim et al., 2016), the United States (Hanna et al., 2019), and the Republic of Côte d’Ivoire (Scheim et al., 2019). Consequently, mental health issues do not result from gender incongruence and stress/rejection/discomfort experienced by the individuals alone but are possibly further promoted by the binary-gendered thinking and treatment routines of the healthcare systems as they exist in most societies around the globe.

Interestingly, the question why trans^∗^gender people have increased comorbidity rates can still be considered unanswered (Reisner et al., 2016). Some authors refer to the model of internalized homonegativity in order to explain increased risk and high prevalence of mental comorbidities in trans^∗^gender people (Bockting et al., 2013, Bockting, 2015; Breslow et al., 2015). Internalized homonegativity describes how non-heterosexual people internalize socio-culturally predetermined negative attitudes and images (Göth and Kohn, 2014). This model is in line with societies’ heteronormativity as it explains how predominant socio-culturally norms can lead to self-pathologizing (Rauchfleisch et al., 2002; Günther et al., 2019) which in turn can cause psychological distress and may finally result in mental health conditions (Bockting et al., 2013; Breslow et al., 2015; Perez-Brumer et al., 2015; Scandurra et al., 2018). This internalization process can be applied correspondingly to trans^∗^gender persons inasmuch as gender identities are conceived of as stable, binary and invariant personality traits. Accordingly, this can be conceptualized as internalized transphobia (Bockting et al., 2013; Bockting, 2015; Breslow et al., 2015). The notion that mental comorbidities solely arise due to gender incongruence and dysphoria therefore seems decidedly too one-dimensional, ignoring the underlying complexity.

### The Evolution and Current Healthcare for Trans^∗^gender in Germany

Trans^∗^gender healthcare in Germany has a centennial history already. In 1922, the German sexologist Magnus Hirschfeld, founder of the first Institute for Sexology, carried out the worldwide first sex reassignment surgery in Berlin (Bhinder and Upadhyaya, 2021). In the post-war German society, the situation of trans^∗^gender persons was recognized only very haltingly. The so-called “transsexual law” (TSG) from 1980 implemented changes of personal and civil status. The law since required trans^∗^gender persons to undergo surgical alteration of their genitals in order to have key identity documents changed. This was declared unconstitutional only in 2011.

Besides the legal framework there were no regulations for medical and psychotherapeutic healthcare for trans^∗^gender people whatsoever until the publication of the German Standards for the Treatment and Diagnostic Assessment of Transsexuals (1997) (Nieder and Strauß, 2015). These standards provided temporal and diagnostic frameworks and concrete guidelines according to which gender-affirming procedures may take place. Stemming from the desire to enable trans^∗^gender people to follow a self-determined and individualized transition, the new S3 guidelines from 2018 [“Gender incongruence, gender dysphoria, and trans health: S3 guideline on diagnosis, counseling, and treatment” (Arbeitsgemeinschaft der Wissenschaftlichen Medizinischen Fachgesellschaften [AWMF], 2019)] have been developed in collaboration with experts and interest groups. In contrast to the precursor from 1997, the new guidelines take a less directive and more participatory approach (Nieder and Strauß, 2019). Hence, treatment seekers and treatment providers are now able to find individual solutions together on equal terms. Access restrictions should no longer exist. Thus, gender-affirming hormone treatment can already be used after diagnosis, at the beginning of the transition. Psychotherapy should no longer be a prerequisite for gender-affirming therapy but should accompany the transition and promote self-acceptance and stability (Nieder and Strauß, 2019). However, the report guidelines of the medical service of the health insurance funds (MDS) contradict the S3 guidelines by continuing to set strict framework conditions for the treatment costs to be covered by the public health insurance funds. Also, the guidelines for the diagnosis of trans^∗^gender criteria from the ICD-10 catalog are less flexible and more stigmatizing than the S3 guidelines. Trans^∗^gender is coded as “transsexualism” (Graubner, 2013). There, the main criterion is the desire of a person to belong to the binary opposite gender. This may include the desire to change sex characteristics (primary or secondary) and to be recognized as belonging to this gender. The desire must be constant for 2 years and must not result from mental disorder. The ICD-10 defines transsexualism as a disorder, subclassified in the section of disorders of adult personality and behavior (Graubner, 2013).

Therefore, practitioners in Germany find themselves in a field of tension between the prevailing strict conditions imposed by health insurance and the ICD-10 catalog and attempts to loosen the regulations in accordance with the individual needs of trans^∗^gender people. This also explains ambivalent reactions and uncertainties on the part of the practitioners to the S3 guidelines (Nieder and Strauß, 2019). In this constellation, it is expected that the new ICD-11 catalog 2022 will bring further change, as transsexualism will be coded in the section “Conditions affecting sexual health,” thus separating trans^∗^gender from somatic or mental illness (Jakob, 2018). This was already successfully implemented in the Diagnostic and Statistical Manual of Mental Disorders-V (DSM-V), according to which it is only possible to speak of a disorder when there is relevant suffering due to the gender incongruence (dysphoria) (American Psychiatric Association, 2013). According to MDS the assessment instructions will have to be revised after ICD-11 has been established.

**FIGURE 1:**
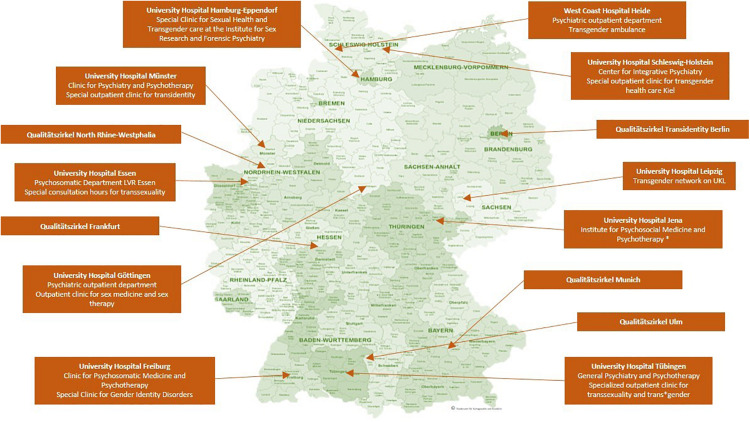
Institutions providing specialized trans^∗^gender healthcare in Germany. The map shows the location of clinics and regional associations of multidisciplinary healthcare specialists (“Qualitätszirkel”) that offer specialized trans^∗^gender healthcare in Germany, without claiming to be exhaustive. ^∗^: According to the clinic‘s website only expert opinions are issued. However, this is listed differently on the website of https://transmann.de. © Bundesamt für Kartographie und Geodäsie.

Trans^∗^gender healthcare in Germany is provided in different institutions. Usually, medical services are provided in private practices. In addition, interdisciplinary healthcare supplies are available via outpatient care, such as the regional “Qualitätszirkel.” These are regional associations of multidisciplinary trans^∗^gender healthcare specialists. There are hardly any centers that offer multiprofessional treatment. The interdisciplinary care center at the University Hospital of Hamburg plays a pioneering role in this area. Some university hospitals offer specialized consultation hours, such as the specialized outpatient clinic for transsexuality and trans^∗^gender in Tübingen, which was established in October 2020. This service is primarily aimed at trans^∗^gender people before and during transition. To the best of our knowledge, there are no central registers for medical services for trans^∗^gender people. Online, there are lists of addresses maintained by interest groups. Figure 1 depicts the institutions providing treatment in Germany and the “Qualitätszirkel” (individual practices or clinics that only cover somatic needs are not listed). They offer various services: psychotherapeutic support, indication letters, medical reports to the TSG and partly interdisciplinary services. Healthcare services offered to trans^∗^gender persons are covered by the health insurance and thus are covered publically, as was decided in 1987 by the Federal Social Court, the Bundessozialgericht (BSG 3 RK 15/86). However, letters of indication from experts are necessary in order that services (e.g., hormonal treatment, surgery) are covered by the health insurance.

### How Does the Healthcare System Understand Trans^∗^gender Nowadays?

Trans^∗^gender people experience incongruence between the sex assigned at birth and their gender identity. Sex assignment is based on the external genital, which are usually defined in medical literature as indicators of the so-called biological sex. To avoid classifying non-binary gender identities as a deviation from the biological sex, the terminology “assigned gender” or “assignment gender” seems more suitable than the term “biological sex” (Günther et al., 2019). Gender identity describes a person’s certainty and conviction to belong to a certain gender (Eckloff, 2012). This develops during the course of a person’s life and is shaped by biological and social conditions equally (Göth and Kohn, 2014). In trans^∗^gender people, gender identity does not develop in accordance with the assigned sex; the result can be a binary or a non-binary form of gender identity: Binary trans^∗^gender indicates that individuals experience themselves as belonging to the binary opposite gender (i.e., transman or transwoman). However, there are also people who feel they belong to neither the female nor the male gender and/or experience their gender on a continuum between the sexes (Günther et al., 2019).

In terms of prevalence rates in European countries, similar rates have been reported, always indicating a slightly higher prevalence rate for trans^∗^women. The prevalence of the ICD-10 diagnosis of transsexualism is estimated at 1:12000 for trans^∗^women and 1:30000 for trans^∗^men in Germany (Schneider et al., 2007). In Belgium, 1:12900 trans^∗^women have undergone gender-affirming surgery, while in men this ratio is approximately 1:33800 in trans^∗^men (De Cuypere et al., 2007). Netherlands show similar prevalence rates (1:11900 for trans^∗^women and 1:30400 for trans^∗^men) (Bakker et al., 1993). However, an increase in prevalence has been reported in several countries: in Germany, for example, a 2.6-fold increase in the number of inpatients who were diagnosed with a gender identity disorder between 2000 and 2014 has been reported (data of the German Federal Statistical Office) (Brunner et al., 2017). Brunner et al. (2017) discuss the increased amount of informational martials and the facilitated access to gender-affirming therapy as a cause of the reported increase in prevalence. Whether and how destigmatization of trans^∗^gender individuals further contributes to the increased prevalence rates needs to be investigated. Unfortunately, standardized prevalence rates of trans^∗^gender individuals are rarely to be found (Collin et al., 2016), as different definitions of trans^∗^gender samples lead to different results in prevalence, obscuring the systematic investigation. Furthermore, the prevalence might be underestimated, as not all trans^∗^gender persons seek gender affirming therapy (De Freitas et al., 2020). After the introduction of the new ICD-11, it should be possible to record comparable prevalence rates of the diagnosis gender dysphoria instead of transsexualism.

### The Healthcare System’s Influence on the Emergence and Maintenance of Suffering of Trans^∗^gender People Focused on the Situation in Germany

The German medical system has institutionalized stigmatization of non-binary people, which has to be especially considered a substantial factor of trans^∗^gender persons’ healthcare situation. This mainly applies to non-trans^∗^gender specific medical care, but also partly to trans^∗^gender healthcare. The variety of experiences of discrimination within the healthcare system have already been pointed out (Franzen and Sauer, 2010; Grant et al., 2011; LesMigras, 2012; Bradford et al., 2013; Roberts and Fantz, 2014; Günther et al., 2019). However, since discrimination refers to distinctions that lead to, produce, or give rise to disadvantage (Scherr et al., 2017), it often seems more appropriate to speak of stigmatization in the context of trans-specific healthcare in Germany. Stigmatization means the designation and marking of a deviation from a norm which is given or desired within a society (Goffman, 1963). Stigmatized persons are denied the status of a normal member of society because of an attribution of characteristics marked as a deviation. Institutional stigmatization occurs within social systems or organizations, where routines in communication and actions perpetuate “normality,” which force the presentation and treatment of deviations from this norm as explicit deviations. Trans^∗^gender people experience this institutional stigmatization in modern medicine in Germany and worldwide (Franzen and Sauer, 2010; Fuchs et al., 2012; LesMigras, 2012).

In itself, the structure of the healthcare system in Germany can be experienced as exclusionary by trans^∗^gender individuals: Identification documents, such as health insurance cards, may not match the gender, cause confusion in providers and can lead to misgendering which in turn is experienced as stigmatizing (Roberts and Fantz, 2014). In the context of medical treatment in Germany, they presumably experience not so much discriminatory disadvantage as invalidation of their gender identity. Günther et al., suggest that exposition to the healthcare system may trigger internalized transphobia among trans^∗^gender people, due to the fact that it occasionally puts the trans^∗^gender individual under pressure to legitimize their own gender identity (Günther et al., 2019).

Because of experienced and/or feared stigmatization, some people are not willing to utilize the medical system. Studies from different countries show that the use of the healthcare system in trans^∗^gender people is reduced due to fear of discrimination (Bauer et al., 2014). In the US-American “national transgender survey” stigmatization experiences of trans^∗^gender persons were documented. One of the key findings reports a high likelihood of discrimination if the medical provider knows about their patients trans^∗^gender identity. They also identify a lack of knowledge by the medical providers, so most of trans^∗^gender people themselves have to inform their doctors about trans^∗^gender healthcare (Grant et al., 2011). In Germany trans^∗^gender persons report that their experiences with the healthcare system depend on whether their trans^∗^identity remains hidden or becomes visible (LesMigras, 2012). Stigmatizing experiences in the healthcare system are among the most common negative experiences of trans^∗^gender persons in Germany, after discrimination at the workplace (LesMigras, 2012). As a result, the health of this group of people is under-supplied, as they typically leave the health system after negative experiences and seek help elsewhere (Mizock and Lewis, 2008).

Due to the deeply embedded heteronormativity in Germany’s society, it is unsurprising that medical areas that are not primarily oriented toward trans^∗^gender healthcare show an unprofessional handling when they face gender identities that do not correspond to this supposed norm. Correspondingly, a study in North-Rhine-Westphalia (Germany) shows that trans^∗^gender persons were hardly satisfied with their psychotherapeutic support (Fuchs et al., 2012). The same study reveals the administrative and treatment burdens caused by the MDS review procedure. It has also been shown, that the institutional pathologizing of trans^∗^identity is experienced as a tremendous burden by trans^∗^gender people (LesMigras, 2012). As outlined in section “The Evolution and Current Healthcare for Trans^∗^gender in Germany,” the German healthcare system has been developing new ways of dealing with trans^∗^gender healthcare. It is in a transition period between strict regulation and self-determination of the trans^∗^gender community. Studies on the fears and wishes of the trans^∗^gender community for multiprofessional treatment centers (as the one in Hamburg) show that also on the part of the treatment providers this development is being worked on and the offers are being adapted to the needs of the trans^∗^gender community (Eysell et al., 2017).

Trans^∗^gender people depend on the healthcare system as they require medical professionals before, during and after gender affirming therapy. Even after a successful transition, psychotherapeutic and somatic care must be ensured. Due to hormone therapy, trans^∗^gender persons have a different lifetime risk profile for cardiovascular diseases (Aranda et al., 2019; Dutra et al., 2019; Pyra et al., 2020). The risk for sex-hormone dependent cancers is not higher during gender-affirming hormone therapy, but the cancer screening recommendations have to be considered in trans^∗^gender people as well, i.e., prostate cancer screening in transwomen or breast and cervical cancer screening in transmen (McFarlane et al., 2018). Because of this need, it is alarming that the stigmatization in the healthcare system increases the chance for trans^∗^gender people to avoid medical care and balk preventive measures, such as cancer screenings (Günther et al., 2019; Weyers et al., 2021).

In addition, studies show that after gender-affirming therapy, psychological stress can also occur, which may lead to increased suicidality (Rolle et al., 2015; Wiepjes et al., 2020). The lifetime prevalence of suicidality is also affected – amongst other variables – by negative experience with medical providers (Haas et al., 2014).

Psychotherapeutic services should also strive to offer gender-sensitive counseling in order to adequately address internalized transphobia, specific role conflicts, and so forth. The need for specialized counseling usually is not met after transition, as trans^∗^gender persons are constantly confronted with their minority status in a binary, heterosexual environment (Verbeek et al., 2020). Unfortunately, specialized training programs for psychotherapists are hardly established. Since medical professionals are usually not trained in gender-sensitive medicine and may be out of their depth with regard to the healthcare of trans^∗^gender persons, this ongoing stigmatization comes as no surprise. Therefore, gender sensitive medicine must become a part of the medical curriculum. There seems to be an interest on the part of medical students (Turner et al., 2014). Finally, gender sensitive medicine has to be implemented in the standard medical care in Germany (Chase et al., 2014).

### Medical Care Services and Barriers for Trans^∗^gender Individuals in Europe

As we propose, the institutionally co-generated psychological strain on trans^∗^gender persons, promotes comorbidities and further increases economic costs. It seems imperative that stigma-free and need-oriented trans-specific treatment is provided by trained personnel. Only then can we reasonably expect that the psychological distress due to gender dysphoria can be minimized and fused conflicts can be addressed e.g., via psychotherapy. There is evidence for a reduction of distress through access to gender-affirming therapy (Bränström and Pachankis, 2020; Almazan and Keuroghlian, 2021).

The mission statement of the European Professional Association for Transgender Health (EPATH), a sub-organization of the World Professional Association for Transgender Health (WPATH), envisions the establishment of uniform European healthcare for trans^∗^gender persons. By drafting a “standard of care” position paper, EPATH tries to formulate a uniform guideline for trans^∗^gender sensitive health care beyond transition. The guideline furthermore establishes basic principles, addressing medical professionals. There is a consensus that healthcare providers worldwide should adhere to these basic principles, regardless of socio-cultural norms and legal requirements of their respective country. *Inter alia*, EPATH urges medical personnel to treat trans^∗^gender persons respectfully and in a non-pathologizing manner. Access to treatment options should be ensured and medical personnel should be further trained in gender-sensitive medicine (Coleman et al., 2012).

However, uniform and comprehensive care for trans^∗^gender persons is far from guaranteed in Europe, as the legal and medical situation is highly diverse: While some countries have been trying to ensure appropriate treatment of trans^∗^gender persons in the legal and medical domain, trans^∗^gender people in other countries are faced with persecution and discrimination (ILGA Europe Annual Review, 2021d). Apart from that, a legal and medical situation that considers the needs of trans^∗^gender people does not necessarily imply that sufficient medical care is provided or that medical staff are sufficiently informed. While there are specialized treatment centers in many European countries nowadays, trans^∗^gender individuals generally face the problem of long waiting times due to the structural lack of healthcare providers in the area of gender-affirming treatment services. In Netherlands treatment options (i.e., diagnostic classification, subsequent gender-affirming therapy such as hormone therapy, gender-affirming surgery) are offered in health centers (Amsterdam UMC, Groningen UMC and Radboud UMC Nijmegen). Like the center in Hamburg, they provide an interdisciplinary treatment – the so-called “gender team.” However, these centers are far from meeting the demand and a lack of healthcare providers in Netherlands has been pointed out recently (Verbeek et al., 2020). In Belgium, care is also provided in healthcare centers (Belgien Universitär ziekenhuis Ghent, Université libre de Bruxelles and Le centre hospitalier universitaire de Liège), with the center in Ghent offering interdisciplinary care (Elaut, 2014). In contrast to Germany, hormonal treatment in Belgium is already possible during full-time real-life experience (Steinmetzer and Groß, 2008). Here, too, the long waiting times have been pointed out as problematic and as an obstacle to the access of appropriate services (Motmans et al., 2010). Specialized care centers in England, Scotland, and Northern Ireland are listed by Vincent (2018). He points out that trans^∗^gender persons have the longest waiting times of all patients in need of specialized treatment services. In Spain, the healthcare is installed in multidisciplinary gender units in different communities all over the country and the Canary Islands (Gómez-Gil et al., 2019). New healthcare models deviate from the central multidisciplinary gender units, for example by offering gender-affirming healthcare without psychological assessment. These new healthcare models are the subject of controversy, because the decentralization can be considered a missed opportunity: (a) for research and (b) to collected data to evaluate the quality of healthcare (Gómez-Gil et al., 2020a,b).

The procedure for gender-affirming surgery in Denmark is prescribed by the Danish Health Authority and is centralized in three clinics (e.g., The Sexological Clinic, Ringshospitalet Copenhagen) (ILGA Europe Annual Review, 2021c). Italy is a positive example of a publicly accessible database of medical care professionals. On the website https://www.infotrans.it/, published in 2020, trans^∗^gender people can find out about treatment services (ILGA Europe Annual Review, 2021b).

The situation in Poland stands out as a negative example in a discrepancy to the, as not sufficiently marked, but existing care situation in most European countries. There, no medical care for trans^∗^gender persons is guaranteed. In addition, there is talk of a hate campain against the LGBTIQ community (ILGA Europe Annual Review, 2021a).

Overall, healthcare in Europe is taking important steps toward depathologization, and many countries are attempting to establish the requirements of WPATH/EPATH. Worldwide, however, conditions remain poor and self-determination rights are denied to the trans^∗^gender community. In some countries trans^∗^gender persons are still criminalized (e.g., Indonesia, Niger, Malaysia, United Arab Emirates).

### Key Findings

**FIGURE 2:**
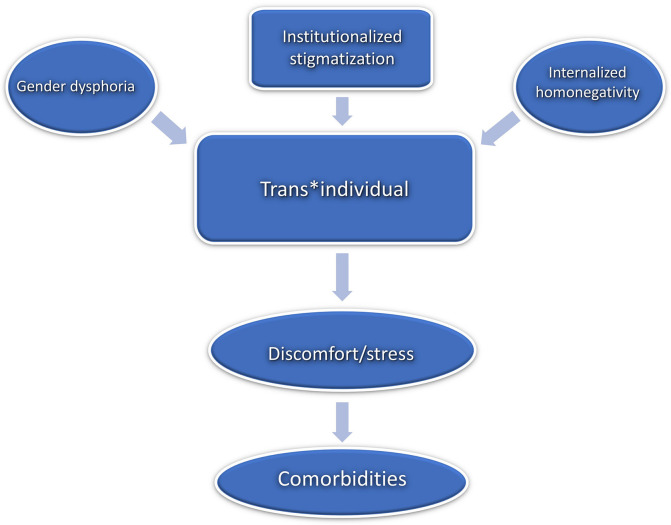
Multifactorial condition of suffering. © Bundesamt für Kartographie und Geodäsie.

The article reviews the medical care situation for trans^∗^gender people nowadays and it provides a more detailed description of the situation in Germany. Three main deficiencies were identified, that are linked to medical care for trans^∗^gender persons in the German healthcare system: (1) A lack of specialized medical care to support transition. Mental comorbidities could be reduced by individualized support during transition. However, this is usually hindered by significant organizational and institutional barriers. Deficits in the structure of specialized healthcare services in Germany and Europe have been pointed out. There is a lack of specialized care offers that ensure a safe place for good care and that alleviate individual suffering through a professionally accompanied transition. (2) A lack of gender-sensitive psychotherapeutic support before and after transition, which could address the trans^∗^gender specific dysfunctional internalization processes in a patient-oriented, professional manner. (3) A lack of sensitivity to special treatment needs in post-transition healthcare. We elaborated that even after transition, a non-discriminatory integration into the healthcare system remains necessary. Due to exclusively binary gender thinking, medicine is prone to institutional stigmatization. Accordingly, trans^∗^gender people are frequently confronted with deficits and hurdles with the safeguard of their health. The multifactorial condition of suffering is modeled in Figure 2.

## Perspective

We see an urgent need for the establishment of comprehensive gender-affirmative healthcare. We propose three starting points: (1) A nationwide structure of specialized treatment centers for trans^∗^gender healthcare is needed. In particular, the problem of unacceptably long waiting times must be addressed. (2) Specific sexual medicine training at an early stage (i.e., at university level during education and later on in specialist training) should lay the groundwork to minimize the institutional stigmatization of trans^∗^gender individuals. (3) Finally, we call for the establishment of psychotherapeutic specialization as well as further education programs to support appropriate treatment of the diverse and multifactorial psychological issues of trans^∗^gender people.

It should be pointed out, that through increased cooperation between medical providers and advocacy groups (e.g., Transgender Europe, TGEU), the European healthcare system can be transformed into a system based on self-determination and informed consent. It is time to face and address the many faceted barriers trans^∗^gender people are facing when confronted with the healthcare system in different European countries (and probably world-wide).

In Germany we see a significant progression within the medical system toward the recognition of the trans^∗^gender community and its needs in the recent years. The implementation of the new S3 guidelines is becoming more and more important and the trans^∗^gender community is becoming more and more involved. Unfortunately, this development has not yet reached all areas. The new ICD catalog in 2022 will be an important step to further improving healthcare of trans^∗^gender individuals. We hope to contribute to establishing improved, gender-sensitive medical care in line with the variable demands of trans^∗^gender people.

## Data Availability Statement

The original contributions presented in the study are included in the article. Further inquiries can be directed to the corresponding author.

## Author Contributions

NG prepared the first draft of the manuscript. All authors contributed to critically revising and editing the content of the manuscript and approved the final version of the manuscript for submission.

## Conflict of Interest

The authors declare that the research was conducted in the absence of any commercial or financial relationships that could be construed as a potential conflict of interest.

## Publisher’s Note

All claims expressed in this article are solely those of the authors and do not necessarily represent those of their affiliated organizations, or those of the publisher, the editors and the reviewers. Any product that may be evaluated in this article, or claim that may be made by its manufacturer, is not guaranteed or endorsed by the publisher.
